# Identifying the unmet information and support needs of women with autoimmune rheumatic diseases during pregnancy planning, pregnancy and early parenting: mixed-methods study

**DOI:** 10.1186/s41927-018-0029-4

**Published:** 2018-07-27

**Authors:** Rhiannon Phillips, Bethan Pell, Aimee Grant, Daniel Bowen, Julia Sanders, Ann Taylor, Adrian Edwards, Ernest Choy, Denitza Williams

**Affiliations:** 1 Division of Population Medicine, School of Medicine, Cardiff University, Cardiff, UK; 2 Centre for Trials Research, Cardiff University, Cardiff, UK; 3 School of Healthcare Sciences, Cardiff University, Cardiff, UK; 4 Centre for Medical Education, Cardiff University, Cardiff, UK; 5 Division of Infection and Immunity, School of Medicine, Cardiff University, Cardiff, UK

**Keywords:** Autoimmune rheumatic disease, Pregnancy, Family planning, Parenting, Infant feeding, Information, Support, Timeline, Qualitative, Visual methods

## Abstract

**Background:**

Autoimmune rheumatic diseases (ARDs) such as inflammatory arthritis and Lupus, and many of the treatments for these diseases, can have a detrimental impact on fertility and pregnancy outcomes. Disease activity and organ damage as a result of ARDs can affect maternal and foetal outcomes. The safety and acceptability of hormonal contraceptives can also be affected. The objective of this study was to identify the information and support needs of women with ARDs during pregnancy planning, pregnancy and early parenting.

**Methods:**

This mixed methods study included a cross-sectional online survey and qualitative narrative interviews. The survey was completed by 128 women, aged 18–49 in the United Kingdom with an ARD who were thinking of getting pregnant in the next five years, who were pregnant, or had young children (< 5 years old). The survey assessed quality-of-life and information needs (Arthritis Impact Measurement Scale Short Form and Educational Needs Assessment Tool), support received, what women found challenging, what was helpful, and support women would have liked. From the survey participants, a maximum variation sample of 22 women were purposively recruited for qualitative interviews. Interviews used a person-centered participatory approach facilitated by visual methods, which enabled participants to reflect on their experiences. Interviews were also carried out with seven health professionals purposively sampled from primary care, secondary care, maternity, and health visiting services.

**Results:**

Survey findings indicated an unmet need for information in this population (ENAT total mean 104.85, SD 30.18). Women at the pre-conception stage reported higher needs for information on pregnancy planning, fertility, giving birth, and breastfeeding, whereas those who had children already expressed a higher need for information on pain and mobility. The need for high quality information, and more holistic, multi-disciplinary, collaborative, and integrated care consistently emerged as themes in the survey open text responses and interviews with women and health professionals.

**Conclusions:**

There is an urgent need to develop and evaluate interventions to better inform, support and empower women of reproductive age who have ARDs as they navigate the complex challenges that they face during pregnancy planning, pregnancy and early parenting.

**Electronic supplementary material:**

The online version of this article (10.1186/s41927-018-0029-4) contains supplementary material, which is available to authorized users.

## Background

When Autoimmune Rheumatic Diseases (ARDs) affect women of reproductive age, this raises a range of issues around family planning, pregnancy, and early parenting [1, 2]. Both ARDs and their treatments can cause problems with fertility, complications during pregnancy, and impact on contraceptive choices [1, 3]. Many women with ARDs will have positive pregnancy and parenting outcomes but there are risks involved [4, 5]. Women with ARDs who are of childbearing age face complex choices about starting (or enlarging) a family now or sometime in the future [6], but they struggle to get enough information and support [2].

Nearly half of pregnancies in Britain are not planned [7]. Choices of contraception can be complicated for women with ARDs, for example the combined progesterone and oestrogen oral contraceptive pill is not recommended for women with more severe forms of Lupus, particularly when they have renal involvement or test positive for antiphospholipid antibodies [3]. Nonetheless, the vast majority of women with rheumatic diseases have viable contraceptive options, including barrier methods, intra-uterine devices and progesterone-only medication [8]. In a survey completed by 212 women of reproductive age who had Systemic Lupus Erythematous in the United States, 97 (46%) women were at risk of unplanned pregnancy (unprotected sex or unreliable method of contraception) in the past three months. A survey in Switzerland (*n* = 170 women) found that around a third of women with inflammatory arthritis who are taking medication that is contraindicated in pregnancy, such as methotrexate and leflunomide, use ineffective or no contraception [9].

In an Australian mixed methods study (*n* = 27), women with Rheumatoid Arthritis (RA) reported that they struggled to find enough information about family planning, pregnancy and early parenting [2]. The study indicated that there was a high demand for more information on the safety of medications during pregnancy and breastfeeding in particular [2]. While Rheumatologists were the primary source for information, women also placed a high value on patient-facing arthritis organisations and on learning from the personal experiences of other women [2]. A systematic review [10] of interventions to improve knowledge and self-management skills around contraception, pregnancy and breastfeeding in women with RA identified only one well designed evaluation of education or self-management focused on pregnancy [11]. In that study, a decision aid for women with Rheumatoid Arthritis to support their decision making about starting (or enlarging) a family improved knowledge about RA and pregnancy and decisional conflict [11]. A further eight studies that were identified in the review of general Rheumatoid Arthritis self-management interventions included a minor component on family planning [10]. Three of these contained information about methotrexate use in pregnancy and/or breastfeeding [12–14], one included a warning about lack of evidence with regard to the safety of biological therapy use during pregnancy [15], three provided advice on relationship, family, and/or sexual issues [16–18], and one involved a discussion about contraception and fertility [19]. Only four of these studies included an outcome measure relevant to family planning or pregnancy [12–14, 19].

More integrated care and better information and counselling around pregnancy and early parenting for women with ARD and other chronic diseases have been recommended [2, 10, 20–23]. However, there is little high quality evidence on how to meet the educational, self-management or broader non-pharmacological health and social care needs of women with ARDs in this context [10]. The objectives of this study were to establish what the unmet information and support needs of women in the UK who have ARDs are during pregnancy planning, pregnancy and early parenting, and to identify opportunities to better meet these needs.

## Method

### Design

We used a mixed methods design, which incorporated a cross-sectional online survey and qualitative interviews with women with ARDs and health professionals. To enable comparisons between this UK study and a previous Australian study [2], we used similar sampling methods and inclusion criteria, and included a modified version of the Educational Needs Assessment Tool [24, 25] to assess information needs.

### Online cross-sectional survey

#### Participants and recruitment

The survey was made available using the Bristol Online Surveys system. The patient survey was advertised through the study website, social media (Twitter & Facebook), via UK arthritis patient organisations (Lupus UK, Arthritis Care, Vasculitis UK), peer-support groups (Facebook groups for people trying to conceive/pregnancy in Lupus and vasculitis), and online networks for parents (Netmums and Mumsnet). We also used Facebook and Twitter advertising systems to promote the study. To facilitate recruitment, we offered the following incentives: a donation of 50p for each questionnaire completed to UK arthritis charities, and an option to enter a prize draw to win a £100 in gift vouchers.

#### Inclusion criteria

Women aged 18–49 years, who have an ARD (i.e. inflammatory arthritis or auto-immune connective tissue disease for which people would normally be under the care of a rheumatologist), and were: planning to become pregnant in the next 5 years; and/or currently pregnant; and/or had been pregnant within the last 5 years; and/or had a child (or children) under 5 years of age.

#### Exclusion criteria

Disease not classified as an ARD (e.g. joint hypermobility, fibromyalgia).

#### Measures

##### Information needs

A modified version of the Educational Needs Assessment Tool (ENAT) [24, 25] was used, which is a validated measure with 39 items to assess educational needs in seven domains; pain management, movement, feelings, the arthritis process, treatment from health care professionals, self-management and support from others. An international study [24] demonstrated that the ENAT is a valid tool for identification of information needs relating to rheumatic diseases, with high internal consistency. The items are scored on a five point Likert scale, providing total score ranging from 0 (lowest educational need) to 156 (highest educational need) [24]. While uploading the modified ENAT for use in the current study to the online survey software, one response category (‘fairly important’) was omitted, and consequently items were scored on a four-point scale: 1-not at all important, 2-a little important, 3-very important, 4-extremely important. To retain as much comparability as possible with previous studies, the individual item scores were transformed from a four point (1–4) to a five point (0–4) scale prior to calculation of the total score, so that its overall range would correspond with the original ENAT (0–156). The subscale total scores were Rasch transformed to provide interval rather than ordinal level data [25].

Additional items were included in the information needs section of the survey, using the four-point Likert scales to assess information needs in relation to: sex and relationships, contraception, preparation for pregnancy, how to increase chances of getting pregnant naturally, fertility treatments, options for giving birth, managing pain during childbirth, and breastfeeding. These items were developed by the research team based on the educational needs identified through previous studies [2, 10, 11], and guided by two Patient and Public Involvement representatives (both women with young children who had ARDs) who highlighted which issues were most important to them from a patient perspective. The patient representatives also requested items be included on the use of transcutaneous electrical nerve stimulation (TENS) as this may be useful for pain management, and how they could get access to their test results, which could prove difficult between appointments. The additional items from the family planning, pregnancy, and early parenting were scored separately from the original ENAT items and were not included in the total ENAT score.

##### Disease-related quality of life

This was assessed using the Arthritis Impact Measurement Scale Version 2 Short Form (AIMS2-SF) [26]. The AIMS2-SF is a validated 26-item measure with five factors; physical symptoms, mobility, role (work), social interaction, and affect. The AIMS2-SF has similar psychometric properties to the AIMS2, good test-retest reproducibility and sensitivity to change. Items are scored on a 5-point Likert scale from 0 to 4, and in each component scores are normalised so that they range from 0 (perfect health to 10 (worst possible health) [26]. Thus, higher scores indicate a greater impact of arthritis on each of these domains.

##### Lived experience and expressed support needs

Using open-response items, participants were asked what they found: i) most challenging; ii) most helpful, and; iii) what support they would have wanted while planning a family, being pregnant, or having young children. Participants were asked whether they were currently having, had previously had, or wanted the following types of support: access to a health professional to act as their main point of contact and care coordinator; physiotherapy; opportunity to talk to other people with similar experiences and to get advice (i.e. peer-support); talking therapies (e.g. counselling, Cognitive Behavioural Therapy); alternative and complementary therapies (e.g. acupuncture, aromatherapy, herbal remedies). These items were developed by the research team based on sources of information and support for women with long-term illnesses while they are building a family that were identified in the literature [2, 4, 10, 21]. The items were reviewed by two patient representatives to assess relevance, clarity and acceptability of the questions.

##### Clinical and demographic information

The survey included questions on type of ARD (drop down list); years since onset of ARD; current medication (drop down list); co-morbidities (open text), and; family situation (currently pregnant, planning to try to get pregnant within the next 5 years, and/or had a pregnancy in the last 5 years, had children already, and if so, how many and what their ages are). Demographic data were collected on date of birth, highest educational qualification, geographical location (postcode), marital status, ethnicity, and current employment status.

#### Survey data analysis

Analysis of the quantitative data was carried out using SPSS v23. Analysis was primarily descriptive, providing an overview of the information and support needs. To identify differences in information needs (ENAT) and quality of life (AIMS2-SF) of women by family status (had or did not have children) and disease group, independent t-tests were carried out, and 95% confidence intervals were calculated. Between-group differences in support received/desired, which were binary categorical variables, were calculated using Chi-square tests. To ensure that there were sufficient numbers for analysis, and due to the differences between rheumatic diseases in disease processes and pregnancy outcomes [1, 4, 5], diseases were broadly categorised as: inflammatory arthritis (rheumatoid arthritis, psoriatic arthritis, ankylosing spondylitis, idiopathic juvenile arthritis, and non-specific inflammatory arthritis), connective tissue diseases (Systemic Lupus Erythematous, systemic sclerosis, and non-specific autoimmune connective tissue disease), and vasculitis. The Holm-Bonferroni correction for multiple comparisons can be used on both parametric and non-parametric tests [27]. The Holm-Bonferroni correction included both the t-tests and Chi-square tests as these tests were conducted on the same data set. Using this method, alpha was set at 0.005. Open text data from the survey were coded thematically using an inductive approach to identify frequent, dominant, and significant themes that emerged from the data [28].

### Qualitative interviews

We adopted a person-centered ethos. As women were being asked about emotional and complex issues in this study, a flexible narrative approach was used to encourage them to talk about their ‘lived experiences’ in their own words, focusing on things that were important to them, rather than being guided by a researcher-generated topics [29]. A timeline-assisted method was used, where participants were asked to create a visual representation of their histories before the interview [30]. Women were sent a ‘What to expect’ sheet was posted to participants along with stationary items, which provided guidance on some of the topics that were of interest to the research team (see Additional file 1). The timelines were used as an elicitation tool in interviews to provide cues and prompt discussion [30]. A topic guide was not used by the researchers during the interviews as the objective of the interviews was to learn about the lived experiences of women, rather than to determine the frequency of predetermined events [31]. The use of participatory approaches such as this in qualitative research can empower participants by allowing them to navigate the conversation, increase their level of comfort in discussing sensitive topics, provide positive moments and opportunities for closure, and can thereby improve the quality of data collected [30, 32].

#### Participants

Women who had expressed interest in being contacted for an interview through the survey were purposively sampled based on their family situation. The aim was to achieve a broadly equal representation of women who were: (i) thinking about getting pregnant; (ii) currently pregnant, or; (iii) had young children, so that the views of women who were at different stages of starting a family could be captured. Women were contacted through e-mail or telephone, based on the contact details provided in the survey. Women who expressed interest in being interviewed were sent a study information pack containing participant information sheet, consent form and stamped return envelope.

Healthcare professionals were identified through professional networks (e.g. Welsh Arthritis Research Network) and were purposively sampled for key professional groups who were involved in the care of women with ARDs in primary care (GPs), secondary care (Rheumatologists, Nephrologists, and Nurse Specialists), and maternity and children’s services (Midwives and Health Visitors – i.e. National Health Service nurses who provide advice and assistance to parents with young children). Healthcare professionals who expressed an interest in participating were e-mailed a participant information sheet and consent form.

#### Interview procedure

Interviews were conducted face-to-face at women’s homes, at Cardiff University, or by telephone. For pragmatic reasons, babies and young children were present during some interviews with women, but no other adults were present. Before the interviews, women were sent a resource pack, which included various items of stationary, an exemplar blank timeline template, and some examples of the themes that we were interested in covering during the interview. This encouraged participants to reflect on their experiences and to guide the discussion during the interview. The timelines provided a visual tool to enable women to map out their journey towards starting a family, noting key events and their physical and emotional responses to these. Women who had prepared timelines could use these as prompts for topics they wanted to discuss during the interviews. Women had the flexibility to use a timeline template provided by the research team, generate their own, or tell their story in their own way if they preferred.

Interviews with healthcare professionals were guided by an interview schedule (Additional file 2), which focused on the health professional’s role, challenges in providing care for women with ARDs who are starting a family, and how care could be improved. Visual timelines were drafted by the researcher at the end of the interview to map out what health professionals had talked about in terms of how healthcare services were provided along women’s journeys through pre-conception, pregnancy and early parenting, and to identify at which points extra support might be needed. The timelines were sent to the healthcare professional after the interview for participant validation, to ensure that they captured the conversation accurately, and healthcare professionals were encouraged to amend the timelines if needed.

Interviews were carried out by Denitza Williams PhD (DW) and Bethan Pell BSc (BP). DW was a post-doctoral researcher and BP a research assistant at the time the interviews were carried out, and both researchers are female. Both researchers had previous experience of carrying out qualitative interviews, and were provided with additional training and supervision in study specific procedures by the lead author (Rhiannon Phillips, PhD) and qualitative lead for this project (Aimee Grant, PhD). The interviewers had no relationship with the participants prior to the interviews and had no prior knowledge of their goals or characteristics, other than the participant information pack provided as described above. Where participants asked the interviewers about their background during interviews, the interviewers explained that they were researchers that were not from a medical background, nor were they experts in rheumatic diseases, but rather that they were interested in hearing about women’s experiences to inform further research on better meeting their information and support needs. Interviews were audio-recorded and interviewers made field notes as soon as possible after interviews. The interviewers requested a copy of the completed versions of the timelines to provide context during the analysis, although this was voluntary. Transcripts were not returned to participants for comment and participants did not comment on the findings. Health professionals were given an opportunity to review and comment on the timelines produced by the researcher to summarise their discussions. No repeat interviews were carried out.

#### Qualitative analysis

All interviews were audio-recorded and transcribed verbatim. The data were analysed thematically using a hybrid approach of inductive and deductive analysis, based primarily on social phenomenology [33]. The analysis focused primarily on the data-driven process of understanding how people make sense of and interpret the phenomena of their everyday world [33]. The deductive component of the analysis was far less pronounced in this study, seeking only to identify themes relating to information and healthcare needs of this and similar populations that had been highlighted in previous research [1, 2, 21]. NVivo V10 was used to facilitate analysis. DW carried out the data coding. Our protocol did not include dual coding of the data. Instead we used regular qualitative research team meetings to discuss data production, the development of the coding framework and data analysis, with each member of the qualitative research group (DW, BP, RP, AG) adding their own unique perspective to the analysis through these meetings. This approach has been identified as appropriate in qualitative research [34]. We were guided by the concept of ‘information power’ [35] rather than ‘saturation’; the research team judged the sample to provide a sufficient depth and range of knowledge to meet the study objectives.

## Results

### Survey findings

The online survey was completed by 131 women. Two of these had diagnoses that were not classified as ARDs and one did not provide information on her diagnosis, so 128 responses were included in analysis. Demographic and clinical characteristics of participants are shown in Table 1. There were no statistically significant differences between women who had children already and those who did not for any of the AIMS2-SF disease related quality of life domains. Women with inflammatory arthritis reported a greater impact of their disease on their physical mobility than those with connective tissue disease (mean difference 0.89, 95%CI 0.23 to 1.56, *p* = 0.009) or vasculitis (mean difference 1.23, 95%CI 0.36 to 2.09, *p* = 0.006), but no other differences were found between disease groups for disease related quality of life.

**Table 1:** Clinical and demographic characteristics of survey and interview participants

		Survey (*n* = 128)	Patient interviews (*n* = 22)
Variable	Category	Number (%)	Number (%)
Primary diagnosis	Systemic Lupus Erythematosus	42 (32.8)	7 (13.6)
	Rheumatoid Arthritis	23 (18)	3 (31.8)
	Vasculitis	23 (18)	6 (27.3)
	Non-specific inflammatory arthritis/connective tissue disease	18 (14.1)	4 (18.2)
	Idiopathic Juvenile Arthritis	9 (7)	1 (4.5)
	Psoriatic Arthritis	7 (5.5)	1 (4.5)
	Other ARD	6 (4.7)	0 (0)
Duration of illness	Up to 1 year	6 (4.7)	2 (9.1)
	1 to 5 years	41 (32)	6 (27.3)
	More than 5 years	79 (61.7)	14 (63.6)
	*Missing data*	*2 (1.6)*	*0 (0)*
Family situation: (number and % responding ‘yes’)	Have children already	71 (55.5)	13 (59.1)
	Thinking about getting pregnant in the next five years	77 (60.2)	7 (31.8)
	Currently trying to get pregnant	9 (7)	2 (9.1)
	Currently pregnant	8 (6.3)	2 (9.1)
	Have been pregnant in the last 5 years	63 (49.2)	11 (50)
Education	Have a university degree	71 (55.5)	16 (72.7)
Employment status	Full time paid work	51 (39.8)	8 (36.4)
	Part time paid work	38 (29.7)	11 (50)
	Unemployed & seeking work	4 (3.1)	0 (0)
	Not employed & currently not seeking work	25 (19.5)	2 (9.1)
	In full or part time education	6 (4.7)	1 (4.5)
	Rather not say	4 (3.1)	0 (0)
Relationships	Married, civil partnership, or living together	107 (83.6)	18 (81.8)
	Other	21 (16.4)	4 (12.2)
	*Missing data*	*2 (1.6)*	*1 (4.5)*
Ethnic group	British, English, Welsh, Scottish, or Irish	114 (89)	19 (86.4)
	Other: non-European	12 (9.4)	2 (9.1)
	Other: European	2 (1.6)	0 (0)
	*Missing data*	*1 (0.8)*	*1 (4.5)*
		Mean (SD)	Mean (SD)
Age	Range: 21 to 48 years	32.75 (6.1)	33.86 (5.3)
Disease-related Quality of Life (AIMS2-SF normalised scores)	Physical	3.52 (1.8)	2.56 (1.38)
	Symptoms	5.41 (3.22)	4.24 (3.51)
	Affect	4.57 (2.4)	4.12 (2.42)
	Social	5.74 (1.76)	6.02(1.64)
	Role	7.79 (3.0)	7.29 (2.80)

Descriptive statistics for information and support needs are shown in Table 2 for all participants, and for those who already have children compared with those do not have children. Women who had children already had higher information needs in the ENAT movement domain than those who did not have children. Information needs relating to the reproductive health items were higher across the board for women who had not yet had children compared with those who had children, with the exception of the sex and relationships item. No statistically significant differences were found in information and support needs by disease group (inflammatory arthritis, connective tissue diseases, or vasculitis).

**Table 2:** Information and support reported in the online survey (*n* = 128)

Variable		All (*n* = 128)	Women who have children (*n* = 71)	Women who don’t have children yet (*n* = 57)	Between group comparisons for women who already have vs. don’t have children		
Information needs		Mean (SD)	Mean (SD)	Mean (SD)	Mean difference	95% CI	*P* value
ENAT (Rasch-transformed scores)	Pain	15.4 (4.94)	16.3 (4.84)	14.2 (4.84)	2.13	(0.42 to 3.84)	0.015
	Movement	13.7 (4.47)	14.6 (4.30)	12.4 (4.40)	2.23	(0.69 to 3.77)	0.005
	Feelings	10.8 (4.44)	11.1 (4.51)	10.5 (4.38)	0.63	(−0.95 to 2.02)	0.431
	Arthritis	20.1 (6.49)	20.3 (6.36)	19.8 (6.48)	0.50	(−1.77 to 2.76)	0.666
	Treatments	18.2 (8.36)	19.1 (8.15)	17.1 (8.56)	2.02	(−0.94 to 4.97)	0.180
	Self-help	16.3 (6.05)	16.3 (6.05)	16.3 (6.11)	−0.05	(−2.20 to 2.10)	0.963
	Support	10.6 (3.41)	10.7 (3.34)	10.4 (3.52)	0.33	(−0.88 to 1.54)	0.587
	Total	104.9 (30.18)	108.2 (29.20)	100.6 (31.14)	7.51	(−3.12 to 18.15)	0.164
Reproductive health information needs (single items, range 0–4)	Sex and relationships	1.8 (1.44)	1.5 (1.33)	2.2 (1.52)	−0.67	(−1.17 to − 0.16)	0.01
	Contraception	1.7 (1.66)	1.3 (1.56)	2.2 (1.66)	−0.93	(−1.50 to − 0.35)	0.002
	Preparing for pregnancy	2.2 (1.76)	1.3 (1.65)	3.4 (1.02)	−2.12	(−2.62 to −1.63)	< 0.001
	Increasing chances of pregnancy naturally	2.1 (1.74)	1.2 (1.60)	3.4 (1.02)	−2.06	(−-2.56 to −1.55)	< 0.001
	Fertility treatments	1.6 (1.71)	0.8 (1.35)	2.7 (1.53)	−1.90	(−2.40 to − 1.39)	< 0.001
	Options for giving birth	2.1 (1.81)	1.2 (1.68)	3.2 (1.38)	−1.89	(−2.44 to − 1.34)	< 0.001
	Managing pain during childbirth	2.0 (1.79)	1.2 (1.65)	3.0 (1.43)	−1.79	(−2.34 to − 1.24)	< 0.001
	Breastfeeding	1.9 (1.75)	1.3 (1.73)	2.6 (1.51)	−1.26	(−1.84 to −0.68)	< 0.001
Support needs		Yes: n (%)	Yes: n (%)	Yes: n (%)	Chi square		P value
Previously had/ currently having	Care planning	73 (57.0%)	45 (63.4%)	28 (49.1%)	2.62		0.111
	Care co-ordination	62 (48.4%)	33 (46.5%)	29 (50.9%)	0.245		0.722
	Peer-support	41 (32%)	19 (26.8%)	22 (38.6%)	2.034		0.184
	Physiotherapy	65 (50.8%)	39 (54.9%)	26 (40.0%)	1.098		0.374
	Talking therapies	41 (32%)	25 (35.2%)	16 (28.1%)	0.741		0.448
	Alternative/complementary therapies	30 (23.4%)	18 (25.4%)	12 (21.1%)	0.326		0.676
	Practical help with daily activities	24 (18.8%)	19 (26.8%)	5 (8.8%)	6.716		0.012
Would like this if available	Care planning	59 (46.1%)	30 (42.3%)	29 (49.2%)	0.946		0.375
	Care co-ordination	67 (52.3%)	40 (56.3%)	27 (47.4%)	1.020		0.374
	Peer-support	80 (62.5%)	48 (67.6%)	32 (56.1%)	1.773		0.202
	Physiotherapy	53 (41.4%)	31 (43.7%)	22 (38.6%)	0.334		0.592
	Talking therapies	67 (52.3%)	37 (52.1%)	30 (52.6%)	0.003		1.000
	Alternative/complementary therapies	72 (56.3%)	41 (57.7%)	31 (54.4%)	0.145		0.723
	Practical help with daily activities	66 (51.6%)	39 (54.9%)	27 (47.4%)	0.724		0.477

### Qualitative findings

Of the 128 survey participants, 118 (92.2%) provided a response to one or more of the open-text questions. Twenty-two out of 88 women approached (25%) took part in interviews. Six women were interviewed face-to-face and 16 were interviewed by telephone. Interview duration ranged from 20 to 85 min, with a mean duration of 48 min for telephone interviews and 64 min for face-to-face interviews. A higher proportion of interview participants had a university degree (72.7% vs. 55.5%) and were in employment (either full or part time) (86.4% vs. 69.5%) than those who were not interviewed. Women who took part in an interview also had a lower AIMS-2 impact of arthritis on physical functioning score than those who did not (95% CI 0.37, 1.95, *p* < 0.005). Three of the women interviewed produced visual timelines, while 12 had prepared notes, prompts, diagrams, or brought along medical records to use in the discussion. Seven healthcare professionals of 25 (28%) invited to interview were recruited. These were two midwives, one health visitor, two consultant rheumatologists, one general practitioner, and one nephrologist. Only one of the health professionals suggested minor changes to the timeline visual produced following their interview to accurately reflect their views.

Thematic analysis of the three sources of qualitative data – the open-text survey items, interviews with women, and interviews with health professionals - revealed three overarching main themes: information needs, multi-disciplinary management, and accessing support. The three main themes and 14 sub-themes that emerged for the three sources of data are summarised in Table 3, and are discussed below.

**Table 3:** Summary of key themes from survey open text questions, interviews with women with ARDs, and interviews with health professionals

Main themes	Sub-themes	Exemplary quotes	Survey responses (*n* = 118)	Interviews with women (*n* = 22)	Interviews with health professionals (*n* = 7)
Information needs	Timing of information & planning	*“It has to be planned because at the moment I can’t just have a child you know, and that doesn’t help because like most people’s family they aren’t planned you know, because I need to find someone who is willing for that as well”* (P2, vasculitis, no children)	Timescales involved with planning a pregnancy were challenging for women: i.e. changing medication a long time in advance of trying to conceive, needing to wait until condition is stable enough to conceive. Risk of unplanned pregnancies & what to do if this happens was a concern. The importance of receiving a timely diagnosis was highlighted.	Women wanted timely, high quality and accessible information. Women recognised that starting a family is a complex process requiring planning. They felt that information about planning a family should be presented and discussed from the point of diagnosis. Some women expressed a need for information about the alternatives to pregnancy, e.g. adoption.	There is an unmet need for quality, timely, written information for women. Pregnancy needs to be planned carefully.
	Disease activity & safe disease management	*“I’ve got my own kind’ve own concerns about you know being at a point where I’m well enough to become pregnant, being well during the pregnancy, can I stay well enough for 9 months that I don’t need extra medication that would impact on the pregnancy?”* (P10, vasculitis, no children)	Women wanted high quality condition specific evidence and advice on pregnancy with ARDs, including information on: the impact of reducing or changing medication while staring a family on disease activity; flare-ups, whether their condition was hereditary, and; what the implications of changes in serum antibody activity were for conception and pregnancy outcomes.	Several women had concerns about unpredictability of ARD following medication change and keeping ‘well’ long enough to conceive. The safety of medications during pregnancy and breastfeeding were often discussed by women, as they felt they had insufficient information about this.	Secondary care physicians felt that women need to be in good physical state with well-managed ARD when trying to conceive.
	Miscarriage	*“You never prepare yourself for having a miscarriage, but we were working on the basis that if I had a successful pregnancy it’s a bonus because the odds were against us as the doctors had said”* (P16, non-specific inflammatory arthritis, two children)	Several women expressed concerns about risks, lack of support following miscarriage, not knowing the cause of miscarriages (i.e. ARD related or other factors).	Women talked about concerns about miscarriage risk, the emotional impact of miscarriage, and not knowing the cause of miscarriage.	Secondary care physicians identified that there is a risk of miscarriage associated with some medicines used to manage ARDs, and that women are likely to need more information and support with this.
	Birth choices	*“I feel like I have less options”* (P5, Systemic Lupus Erythematous, no children)	Challenges women faced included coping with premature births, and lack of involvement in decisions about method of delivery.	Women often experienced a lack of information, and expressed a need for collaborative conversations when discussing options for birth.	Need for collaborative conversations during discussions about birth options was highlighted by secondary care physicians and midwives.
	Infant feeding	*“By about 4 weeks my mum was saying please stop breastfeeding and she’d been very pro-breastfeeding, but she could obviously see I was struggling (with mobility and pain), but I marched on and then at 6 weeks I dropped the child”* (P9, non-specific inflammatory arthritis, two children)	Lack of information & evidence about efficacy and safety of medication to manage disease and pain during breastfeeding was identified as a challenge by several women.	Desire to breastfeed baby whilst also being able to manage ARD symptoms, such as impact of disease flare and pain, was often challenging for women. Women felt that there was a need for more awareness about the impact of chronic conditions on breastfeeding amongst midwives/health visitors.	Midwives identified a need to utilise midwifery expertise in supporting breastfeeding through advice on infant feeding as well as positions for holding the baby.
Multi-disciplinary management	Unmet need	*“This time around I think I didn’t get monitored closely enough during the pregnancy really. I think it must be resources and there’s never any communication between rheumatology and the obstetricians”* (P6, psoriatic arthritis, one child)	Despite requiring input from a range of health and social care services, examples of formal multi-disciplinary team input were rare. Poor communication was important to women, who reported receiving inconsistent advice, not being listened to, and not being believed as challenges. Some women were discouraged from getting pregnant by doctors due to their disease.	Several women felt that there was a lack of multidisciplinary management that included secondary and primary care services. Women felt that their clinicians often focused on the management of their disease, and that they did not view them holistically.	Secondary care physicians reported that women who are planning a family were already managed through multi-disciplinary teams, but acknowledged that this might not be available to women ion all areas of the UK.
	Value of multi-disciplinary care	*“And so it’s actually getting everybody that might need to be involved to see it in a more holistic way.”* (HP1, health visitor)	Women valued care from a range of services in addition to rheumatology, including primary care physicians, obstetrics, counselling, physiotherapy, occupational therapy, and midwifery and health visiting services.	Women who received care from a multidisciplinary team with an open line of communication, usually those receiving their treatment at national centres of excellence, found the approach helpful. Women recognised the value of a multi-disciplinary approach, especially input from midwives and health visitors from the early stages of pregnancy onwards.	High level of consensus that multidisciplinary care is needed.
Accessing support	Regional differences	*“So a lot of the things that are available are area specific and not needs specific you know so your need might meet the threshold but be outside of the area”* (HP1, health visitor) m	Travel to specialist services, and ability to access to fertility services were challenging in some areas.	Women acknowledged that there was considerable variation between regions in the availability of services, including social care and psychological support. Some women reported that travel to secondary care/specialist services can be difficult.	Health professionals recognised that pre-conception counseling was not always available due to regional variation. It was also acknowledged that not all regions within the UK have a multidisciplinary set-up.
	Pre-conception counselling	*“There are other areas of where it (pre-conception counselling) is much less developed and those sort of services tend not to be available and the general NHS approach to these patients is much more chaotic and it’s very much up to the patients to try to find out, you know, the advice”* (HP5, secondary care physician)	Though women talked about the need for more information and emotional support, pre-conception counseling services were not specifically discussed.	Pre-conception advice occurred during secondary care appointments, but provision of advice was minimal for most women.	Pre-conception counselling is fundamental in supporting women with ARDs, but is not universally available. There was variance between health professionals in perceptions of who is responsible for pre-conception counselling (primary or secondary care).
	Care planning	*“If I had an appointment made when I was pregnant so we could’ve planned those early weeks and planned a best case scenario and a worst case scenario (…) maybe it would’ve been nicer to have had a more realistic approach to what approach to what I could achieve”* (P9, non-specific inflammatory arthritis, two children)	Concerns about the potential of disease activity flare during pregnancy/post-partum and the management options. Frequency of appointments and lack of co-ordination of care were challenging for women.	Some women had experienced a lack of rapid access/care planning for post-partum flare ups, but several women reported a lack of care planning. Continuity of care was felt to be particularly important during pregnancy and planning for birth. Women reported that they often had to explain their ‘story’ in relation to their ARD and pregnancy due to a lack of continuity of care.	Health professionals felt there was a need for multidisciplinary care planning, including incorporating occupational therapists.
	Social & practical support	*“The health visitor got in touch with a local council with a (…) families team and I quickly got assigned a social worker the social worker (…) she used to take children (…) to school, she’d bring them in the afternoon and she would give them to the parents in the evening”* (P14, Systemic Lupus Erythematous, three children)	Social care, support from partners and family, and help with childcare were viewed as being helpful. These were areas where women felt that support needed to be improved, along with more support from employers, financial support, and greater understanding and awareness from social welfare agencies. Accessing healthcare could be challenging when caring for young children.	Social and familial support vital for practical/physical demands of parenting was a prominent theme. Views of partners were important to women in making decisions about building a family.	The provision and availability of social support was discussed by some health professionals. It was felt some social support might be available to help with practical aspects of parenting, such as getting children to school or providing care whilst the mother is attending hospital appointments or during hospital admission.
	Peer-support	*“What you really want is somebody else who’s been through that to say this is how they found it”* (P10, vasculitis, no children) (P	Peer-support and learning from the experiences of others were valued by women. Greater availability of peer-support was identified as an area for improvement.	Women often reflected on the availability of online peer-support due to the lack of disease specific groups available. Women wanted to hear about the experiences of other women with ARDs.	Not discussed.
	Tailoring existing healthcare services for women with ARDs	*“I mean we need more education, I’m thinking about health visitors but we need more education I think about attachment because it’s really key in these things and understanding how if you have, whatever it is really, if something hijacks your care-giving experience as an adult, how that affects the sort of longer term outcomes for the child and the relationship.”* (HP1, health visitor)	Women felt existing services should be improved by: providing more involvement in decisions about their health and building a family; provision of consistent and proactive care, and; specialist midwife/specialist nurse involvement during pregnancy. Good communication, clear advice, being open to questions, compassion, kindness, understanding, encouragement, and honesty from health professionals were viewed as being important aspects of care.	Availability and suitability of mother and baby groups as a traditional form of support was frequently discussed. Women reported that they would attempt to engage in mother and baby groups, but would often struggle to fully participate due to their limited mobility. Some women felt there was a need for specialised mother and baby groups. Some women’s experiences with occupational therapy services were that they did not take into account their role as a mother who needs to look after a child.	During pregnancy, health visitors and midwives felt that clinician training in the management of chronic conditions and their potential impact on the family unit was needed to help facilitate a multidisciplinary approach.
	Psychological support	*“I was again feeling totally useless because I should have been able to drive him (son), I should’ve been able to just get dressed, get him in the car and to the hospital and I could not put one foot in front of the other, I was so tired”* *(P14, Systemic Lupus Erythematous, three children)*	More emotional support and counseling was viewed as a way of improving care. Uncertainty about the impact of disease on pregnancy (and vice versa) and ability to cope with demands of parenting resulted in fear and anxiety. The impact on women’s identity was often discussed, with women wanting to be a ‘normal’ parent and to be seen as a whole person not a disease. Having realistic parenting ideals was perceived to be helpful. Positive aspects of parenting included motivation and sense of purpose.	Women expressed a need for more psychological support. For many women, their ARD led to them feeling restrained by their physical symptoms, and they felt that they were unable to do some of the things that ‘normal’ mothers do.	Health professionals felt that it was important to consider the psychological support needs of women. They felt that support for anxiety and depression was needed due to the specific challenges associated with planning a pregnancy, changing medication, managing a pregnancy and coping with a young child whilst also dealing with ARD symptoms.
	Support with functional symptoms	*“(Daughter) was christened in October 2015 and I couldn’t even hold her, I could not stand up and hold her so when she got christened, I couldn’t even hold my daughter at the font”* (P4, non-specific inflammatory arthritis, one child)	Women expressed concerns about whether they would be ‘well enough’ to cope with caring for young children due to fatigue, exhaustion, lack of sleep, pain and mobility.	Several women found that fatigue, pain, and mobility presented challenges when it came to caring for young children.	Health professionals acknowledged a need for the provision of social and psychological support to help women cope with these symptoms.

#### Information needs

Women reported a range of information needs, which corresponded to five sub-themes: timing of information and planning; disease activity and safe disease management, miscarriage, birth choices, and infant feeding. Both the survey and interview data indicated that women wanted timely, high quality and accessible information about these issues. The safety of medications during pregnancy and breastfeeding were often discussed by women:

*But nobody’s told me what the side effects of steroids are during pregnancy, they just say it’s kind of the safest option really and that they’ll judge it when they get to it depending on how bad I am. So I’m walking into the unknown, I have no idea.*

(P13, Rheumatoid Arthritis, no children)

*The thing that I struggled with is that nobody knows, or seems to know how rheumatology and breastfeeding works and I wanted to take the medication if possible.*

(P6, psoriatic arthritis, one child)

Where pregnancy was not an option, women expressed a desire for information about alternatives such as adoption. Health professionals also recognised that there is an unmet need for high quality, timely, written information for women and that pregnancy needs to be planned carefully.

### Multi-disciplinary management

The importance of multi-disciplinary care was a prominent theme in the interviews with women and health professionals. Two sub-themes were identified within this theme: unmet need for multi-disciplinary care, and the value of multi-disciplinary care. Women’s experiences varied widely, but most felt that there was a lack of well co-ordinated multidisciplinary management between different secondary care departments, as well as primary care, and this could undermine women’s trust.

*I’ve always been the go between, the departments don’t really talk to each other and I’ve many a time been in a position when I, I’ve said to either my GP or my consultant you’ve lied to us because you’re both telling me different things.*

(P13, Rheumatoid Arthritis, no children)

All clinicians felt that multidisciplinary management during pregnancy planning, pregnancy, and early parenting was optimal for achieving the best outcomes for women and their children. While secondary care physicians generally reported that women with ARDs who are planning a family or pregnant are already managed through multidisciplinary teams, it was also acknowledged that not all regions within the UK have a multidisciplinary set-up. For example, rheumatology centres in England were thought to be generally better funded and were encouraged to become centres of excellence, whilst in other regions of the UK (Wales, Scotland and Northern Ireland) this was not necessarily the case.

### Accessing support

Women and health professionals recognised that women needed to access a range of services and support, and seven sub-themes emerged during analysis: regional differences, pre-conception counseling, care planning, social and practical support, peer-support, tailoring existing services for women with ARDs, psychological support, and support with functional symptoms. Regional variation in the availability of services, including multi-disciplinary teams, pre-conception counseling, social care, and psychological support were identified through the survey and interviews with women and health professionals, indicating that there was considerable variability in the services available to women. Travelling in order to receive specialist care was also challenging for women, as was attending frequent appointments when they were also caring for young children. Care planning, social and practical support, and psychological support were recognised by women and health professionals as important aspects of care, but were not always available to women. Women also talked about the importance of peer-support, in particular the ability to learn from the experience of others with a similar disease. However, the health professionals did not discuss peer-support.

While women acknowledged that they had a range of unmet information needs, pre-conception counseling as a service was not discussed in either the survey or interviews. Rather, they accessed what they viewed as being minimal pre-conception advice during their secondary care appointments. Health professionals felt that as well as the provision of good quality written information, women would need face-to-face discussions with health professionals because of the complexity of the disease and associated medications, and felt that pre-conception counselling would be needed. There was a discrepancy between different health professionals’ views of which service should offer pre-conception counseling. Midwives felt that it should be conducted by GPs and/or sexual health clinics, whilst GPs and secondary health professionals felt that it should occur in secondary care. In particular, secondary care physicians felt that specialist nurses were best placed to offer pre-conception counselling.

*I think, you know doctors are fine at giving the sort of sciencey side but I think patients are much more likely to open up to specialist nurses (…) Nurses have more time, it’s easier to write that into their job description than it is to write it into doctor’s job description.*

(HP5, secondary care physician)

The need to tailor existing services to meet the specific needs of women with ARDs emerged as a theme. For example, women reported that they would attempt to engage in mother and baby groups, such as baby massage, but would often struggle to fully participate due to their limited mobility.

*Because of course it was a baby massage course the baby’s on the floor. If I was on the floor I couldn’t get up so the first couple of weeks I went I was sat on a chair leaning over but then I just it started to make my back ache and everything else.*

(P4, non-specific inflammatory arthritis, one child)

Women reflected on the difficulties they experienced, and highlighted the need for a modified group suitable for women with limited mobility.

*So maybe, maybe there’s a way of setting up an arthritic mother’s group, or something like that.*

(P9, non-specific inflammatory arthritis, two children)

Health professionals also felt that it was important to consider the specific support needs of women with ARDs.

*I’m just thinking about the, there’s very specific anxiety and concerns that come for these women in the context of their parenting role having a chronic sort of autoimmune disorder.*

(HP1, Health visitor)

The need for greater awareness and education for a broader range of health professionals who would be in contact with women during pregnancy and early parenting, such as midwives and health visitors, was recognised by women and health professionals.

### Recommendations for improving care from women with ARDs and health professionals

A number of recommendations were made by women and health professionals during the interviews for the improvement of care and support of women during pre-conception, pregnancy and early parenting. These focused on the need for clear and timely information about medication, clear and collaborative communication between clinicians and patients, multidisciplinary management, and increased practical as well as emotional support. Table 4 outlines the general recommendations made by both women and health professionals.

**Table 4:** Recommendations from women with ARDs and health professionals for improving of care and support during pre-conception, pregnancy and early parenting

Recommendations	Women (survey and interview)	Health Professionals
Information and communication	Clear information on medication use during pregnancy planning, pregnancy and breastfeeding	High quality written information: pre-conception, pregnancy, post-partum and pre-conception counselling
	Patient-centered approach. More empowerment and involvement in decisions about medication	The need for a patient-centered approach (shared decision-making) in consultations
Multi-disciplinary management	Multi-disciplinary, proactive and better coordinated care (mainly rheumatology, obstetrics, fertility and mental health services)	More training for health professionals such as health visitors, occupational therapists, and midwives about chronic conditions and their impact
Support	Tailoring of professionally led mother and baby groups to ensure they are suitable for women with a chronic condition which affects mobility	Psychological support provided more widely
	Peer-support & information on the experiences of others in a similar situation.	Provision of social care support
	More practical support, such as agencies that can provide childcare and home help	

## Discussion

The findings of our survey and qualitative research indicated that women with ARDs in the UK have a wide range of unmet information and support needs in relation to pregnancy planning, pregnancy, and early parenting. While some had experienced comprehensive multi-disciplinary care that met their expressed needs, others struggled to get any information or support at all with navigating the complex challenges that they faced during this important time in their lives. Health professionals echoed the views of women in many ways, and they felt that pre-conception counseling and a multi-disciplinary approach to care could be particularly useful.

### Information needs

Our findings, in line with those of Ackerman et al. [2], indicated that with women with ARDs report a broad range of unmet information needs when they are building a family. A modified version of the ENAT was used in the current study. Therefore, caution should be taken in directly contrasting scores for this measure with other studies. Nonetheless, the total ENAT scores in the current study and Ackerman et al.’s [2] study with women with Rheumatoid Arthritis in Australia indicated that the overall need for information in this population is high; the total mean ENAT score was 104.9 (SD 30.18) in the current study, and 97.2 (SD 30.8) in the Ackerman et al. [2] study, with the total ENAT score having a range from range 0 (lowest need) to 156 (highest need). In the current study, the greatest expressed need for information related to information about disease processes and treatments from health professionals. Women also expressed a need for specific information in relation to family planning, conception, pregnancy, and breastfeeding. The information needs of women in our study were similar overall across the different types of rheumatic disease and family status. However, we identified statistically significant differences in the information needs of women who already had children compared with those who did not, with the former requiring more information about managing the physical limitations of their disease, and the latter expressing a greater need for information to prepare them for conception, pregnancy, childbirth, and breastfeeding.

Our qualitative findings from the open text sections of the survey and the interviews with women also indicated that women struggled to get the information and support that they needed. Information about their disease, and specifically how it was likely to affect them (and their children) during pregnancy and early parenting was a prominent theme. Women expressed a need for more information about the safety of using medication during breastfeeding, and expressed concerns about how their disease would impact on their role as a parent. From women’s reports, it seems that they were often falling into the gaps in terms of receiving the right information and support with family planning, pregnancy and early parenting. These issues were seen as being peripheral in secondary care where disease management was the main priority, but neither were women’s needs being met elsewhere via services that women without ARDs would typically access.

### Support needs

The AIMS2-SF scores indicated that ARDs had a wide reaching impact on quality of life for women in all the disease groups. In line with this, women reported valuing a range of healthcare, social care, and community based support for managing their disease, physical and emotional symptoms, and practical aspects of daily living. AIMS2-SF scores were broadly similar to those reported for Rheumatoid Arthritis patients in a trial of needs-based patient education [36]. However, in our study women reported a particularly high impact of their disease on work (role domain mean 7.79, SD 3.0). Functional disability and fatigue have previously been identified as having an impact on the parenting roles of women with Systemic Lupus Erythematous [37]. Women with Rheumatoid Arthritis also report that pain and fatigue impacts on their parenting roles [38]. The dual pressure of work and household/family demands can be challenging for younger women with Rheumatoid Arthritis, but employment has important health, social, and emotional benefits that should not be overlooked [39, 40]. Women with inflammatory arthritis reported a higher impact of their disease on their physical mobility than the other disease groups, which would be consistent with the pain, impaired joint mobility, and decreased aerobic fitness that is characteristic of inflammatory arthritis [41]. Nonetheless, our qualitative data indicated that physical functioning was challenging across disease groups, particularly while caring for young children, and information and support needs reported via the survey were similar for those with inflammatory arthritis, connective tissue diseases (including Systemic Lupus Erythematous), and vasculitis.

Women reported that access to health and social care support (both specialist and community-based) during early parenting was variable and fragmented, with some having difficulty even getting their basic medical needs attended to, while others felt they received excellent multi-disciplinary care.

### Healthcare professionals’ views

The interviews with health professionals in our study highlighted the need for a more coordinated and proactive approach to providing women with the information that they need. Physicians felt family planning should be dealt with in a secondary care setting in this context due to the complexity of these diseases, with specialist nurses being well placed to have these discussions. Midwives and health visitors thought that primary care and family planning clinics were well placed to support women with their family planning decisions. In an Australian Delphi study with rheumatologists, obstetricians and obstetric medicine physicians, and pharmacists, guiding principles for clinical practice were that information delivery needed to be: coordinated; delivered in an appropriate mode and format, at the right time, and tailored to the individual patient; based on best available evidence; delivered by the right health professionals at the right time, and; a non-judgmental approach is required for infant feeding [42].

### Implications for clinicians and policy makers

A more coordinated, holistic, and equitable approach is required to ensure that information and support needs of women with ARDs are met during a time in their lives when they are likely to encounter numerous challenges and complex choices. Tailored support is required by women with ARDs at various stages during pregnancy planning, pregnancy and early parenthood, and these issues should be revisited regularly as women’s circumstances change. More holistic and coordinated care could improve health and quality of life outcomes for women with ARDs and their offspring. The roles of different members of multi-disciplinary teams in supporting women of reproductive age with ARDs need to be considered [42, 43].

High quality, consistent and timely information resources need to be made available on the wide range of issues that affects this population. Clinicians’ interpersonal and communication skills are important, as well as fostering a culture of openness and involvement of patients in decisions. A need for pre-conception counseling for women with long-term limiting illnesses has previously been identified [21]. Having children is a highly emotive issue and it has been suggested that women with ARDs should consult with a clinical psychologist when they are preparing for pregnancy [44]. Women in this study reported struggling with miscarriage in particular, and for many their partner’s role in caring for them and their children was vitally important. Clinical psychology, counselling and family therapy services could provide support women and their families with these issues.

Community based support, including peer-support, practical help with caring for young children, raising general awareness, support with infant feeding, and social care also need to be considered to meet the complex needs of this group. Previous studies of the perceived impact of ARDs on parenting roles, including in Lupus [37], Rheumatoid Arthritis [38] and systemic sclerosis [45], have indicated that pain, fatigue and problems with mobility can have a significant impact on the daily tasks associated with parenting, such as picking up and carrying children, or getting up and down from the floor. These challenges were discussed by several mothers that we interviewed, and they highlighted the importance of community based services, such as Occupational Therapy assessments, social care services, domestic help, and support with childcare. However, mothers reported that these services were often orientated towards the mother’s needs as a disabled person, but did not take into account her role as a mother and the tools and adaptations that might benefit her in caring for her child. The potential to tailor these services so that they take into account the needs of pregnant women and families with young children should be investigated.

The themes identified in this study in terms of unmet information and support needs are similar to those reported in studies carried out in Australia [2, 42] and the United States of America (USA) [37, 38, 45, 46]. However, there are differences between Australia, the USA, and the UK in the organisation and structure of healthcare systems [47]. Our study also highlighted considerable variability in the organisation and availability of healthcare services in different regions within the UK. Consequently, needs and support mechanisms are likely to vary nationally and internationally, and this needs to be taken into account in designing interventions to better meet the information and support needs of this population.

### Strengths and weaknesses of the study

Using a mixed-methods approach enabled analysis of data from different sources (survey, interviews with women, and interviews with health professionals), and from people with a wide range of experiences, to identify a range of gaps in meeting the information and care needs of women with ARDs. The survey was cross-sectional, so the association between reported information and support needs and outcomes could not be assessed. The survey used a combination of validated measures and additional items relating to specific reproductive health information needs and sources of support were included that were developed by the research team in conjunction with patient representatives to highlight areas for further research; as such, the latter were not validated measures. The modified ENAT scores provide valuable information on the unmet needs of patients in this study, but due to differences in the rating scales used, comparison with the original ENAT [24] scores reported in other studies should be treated with caution. Survey participants were self-selecting, and it was not possible to calculate response rate using the online recruitment method. Therefore, we do not know to what extent these findings will generalise beyond the study population.

Our in-depth person-centered qualitative research allowed us to understand more about women’s information needs, why and how they were or weren’t met, and how information and support needs could be better met from the perspectives of women with a range of ARDs and health professionals from a variety of disciplines and settings. However, women who took part in interviews were more highly educated, more likely to be employed, and had lower AIMS2 scores than the overall survey participants, which was indicative of a sampling bias that should be taken into consideration when generalising from the findings. We interviewed health professionals from primary, secondary, and maternity health care services, but we were unable to engage with some important professional groups within the confines of the time and resources available for this study, including rheumatology nurses and obstetricians. The primary reason given by health professionals for non-participation in interviews was lack of time due to other demands.

## Conclusions

There is an urgent need to develop and evaluate interventions for women of reproductive age who have ARDs that will improve the quality of information, promote more collaborative decision making with regard to motherhood and healthcare choices, and re-design health and social care services to provide more accessible, timely, integrated, and holistic care.

## Additional files

Additional file 1: Visual timeline template for interviews with women with ARDs. (PDF 198 kb)

Additional file 2: STAR Study Clinician Interview Topic Guides, V1.2, 14.12.16. (DOCX 22 kb)
