# Supplementary material for: Identifying the unmet information and support needs of women with autoimmune rheumatic diseases during pregnancy planning, pregnancy and early parenting: mixed-methods study
Source: BMC Rheumatol. 2018 Jul 27;2:21. doi: 10.1186/s41927-018-0029-4 (PMC6390539; doi:10.1186/s41927-018-0029-4)
Supplement: Supplementary file 1 — Visual timeline template for interviews with women with ARDs. (PDF 198 kb) [file 41927_2018_29_MOESM1_ESM.pdf]

My STAR Study Interview:
What to expect

Before we come to interview you, we would like you to understand what we would like to talk about, and for you to think about these issues.

The interviews are very informal. We would like you to tell us about your experiences in your own words. **You can chose what you would like to talk to us about**. Depending on your own personal situation, this might include things like:

- What it is like to plan a pregnancy when you have an auto-immune rheumatic disease
- Any care and support that you have received with starting a family
- Difficulty with getting pregnant, or losing a baby
- How your illness affected you during pregnancy
- How your illness affected you after your baby was born
- How things are for you now
- What you think about the future

You do not have to talk about anything that you don't want to. If there is anything you don't want to talk about, please let the researcher know before the interview or at any point during the interview. You are free to stop and end the interview at any point, without giving a reason.

Using the timeline

In some of our previous studies, participants have found it helpful to write some notes in advance, and then to talk through these notes with the researcher. We have enclosed a resource pack with some stationary and worksheets that you can use if you'd like to. **If you would prefer not to write anything down, that's also fine.**

Some women find a timeline useful for noting down some of the important things they'd like to tell us about. We have included a timeline template that you can fill in, which includes some blank boxes. You can write as little or as much as you like. You can also chose to create your own timeline without using the template.

You can use coloured pens or stickers to represent how you were feeling at certain points. You could also focus on how your auto-immune disease affected you at different times. If you would like to do this, you could use this key:

- Pain - red
- Tired – orange
- Mobility problems – blue
- Feeling sick or nausea – yellow
- Medication – green
- Healthcare support – purple
- Informal support from family and friends - pink

If you wish to use any other colours to represent different feelings, experiences and emotions, feel free to use colouring pens or pencils, but please include a key at the top.

If you have any questions about preparing for your interview, please do not hesitate to contact Denitza on 029 20687809 or at STARfamilystudy@cardiff.ac.uk
