# Supplementary material for: Identifying the unmet information and support needs of women with autoimmune rheumatic diseases during pregnancy planning, pregnancy and early parenting: mixed-methods study
Source: BMC Rheumatol. 2018 Jul 27;2:21. doi: 10.1186/s41927-018-0029-4 (PMC6390539; doi:10.1186/s41927-018-0029-4)
Supplement: Supplementary file 2 — STAR Study Clinician Interview Topic Guides, V1.2, 14.12.16. (DOCX 22 kb) [file 41927_2018_29_MOESM2_ESM.docx]

**Additional file 2**

**STAR Study Clinician Interview Topic Guides, V1.2, 14.12.16**

**Prologue**

We are very interested in your experience of caring for women with auto-immune rheumatic diseases. We would like you to think about situations when you have been caring for a women with an auto-immune rheumatic disease who was either planning a pregnancy, pregnant or had recently had a child who was under 5 years old. We really want to understand whether you have faced any challenges caring for women and your perception of the challenges faced by women in this group. We also want know whether you feel that women (and/or clinicians) might need further support or interventions that are not currently available and whether you have any ideas about what might help. Are you happy to proceed?

**ARDs - Ankylosing Spondylitis, Inflammatory Arthritis, Polyangiitis, Psoriatic Arthritis, Vasculitis, Lupus, Wegener’s, Churg-Strauss Syndrome, Rheumatoid Arthritis**

**Taking Consent**

Participants will have been sent an information sheet (V1.1. dated 05.10.16) and copy of the consent form (V1.1, dated 06.10.16) in advance of the telephone interview. For face-to-face interviews, written consent will be obtained. For telephone interviews, verbal consent will be obtained.

- This interview will be audio-recorded. The recording will be treated with the strictest confidentiality and may be listened to by the research team but by nobody else. The recording will not be labelled with your name and any written record or report derived from it will be fully anonymised.
- Are there any questions you would like to ask me before we start?
- *[Complete written or verbal consent as applicable. For verbal consent, confirm that the consent process will be audio-recorded].*

**Your role**

Could you talk me through your role in working with women who have auto-immune rheumatic diseases when they are thinking of starting a family, are pregnant and/or have young children?

Roughly how often do you see women with auto-immune rheumatic disease who are planning a family, pregnant or have young children?

Women sometimes describe starting a family as a journey, from staring to think about having children (or finding out that they are pregnant) through to motherhood. At which points during their journey do you think they would:

- Access your services
- Face challenges
- Need extra support

**Challenges**

In your experience, what are the main challenges in providing care for women with auto-immune rheumatic diseases when they *(as applicable to the interviewee’s role)*:

- Are thinking about whether to start a family
- Are planning a pregnancy
- Are pregnant
- Have young children

Could you talk me through an example of a situation when you were providing care to a women with an auto-immune rheumatic disease which you found challenging?

**Improving the quality of care**

Reflecting on your experiences, what would make the most difference in improving the health and well-being of women with auto-immune rheumatic diseases when they *(as applicable to the interviewee’s role)*:

- Are thinking about whether to start a family
- Are planning a pregnancy
- Are pregnant
- Have young children

Could you give me an example of a case or cases where you felt good care had been provided?

What extra support do you think they might need?

Who do you think could provide that extra support?

Why do you think they would be best placed to provide that support?

What types of intervention would you like to see developed?

Do you think any of the following types of intervention would be useful *(if not previously mentioned)*?

- Information and educational resources for women
- Information and educational resources for health professionals
- Self-management support for women (in groups or one-to-one)
- Case management
- Personalised care planing
- Tools to help patients make complicated decisions about treatments/family planning (e.g. option grids)
- Talking therapies (e.g. counseling/Cognitive Behaviour Therapy)
- Physiotherapy
- Peer/lay led support to share experiences and get advice from other people who have been in a similar situation
- Other?

In what way do you think these interventions will help women with auto-immune disease and/or their health professionals?

**Closing**

Do you have any other comments or questions?

Would you like a copy of our findings?

**Thank you very much for taking part in this interview**
